# The accuracy of pedicle screw placement in thoracolumbar fractures: A comparative study of automated cone-beam CT-based navigation and conventional fluoroscopy

**DOI:** 10.1016/j.bas.2026.107447

**Published:** 2026-09-16

**Authors:** Kjell Alexander Thunes Akre, David Andreas Thomas Werner, Maziar Behbahani, Cecilia Isabella Alexandra Avellan, Knut Harboe, Eric Franssen, Anne Kristin Reve, Clemens Weber

**Affiliations:** a Department of Neurosurgery, Stavanger University Hospital, Stavanger, Norway; b Faculty of Health Sciences, University of Stavanger, Stavanger, Norway; c Department of Orthopaedic Surgery, Stavanger University Hospital, Stavanger, Norway; d Faculty of Medicine, University of Bergen, Bergen, Norway

**Keywords:** Cone-beam computed tomography, Thoracolumbar fracture, Pedicle screw, Spinal navigation, Fluoroscopy, Spine trauma

## Abstract

**Introduction:**

Pedicle screw fixation is a standard technique for stabilizing unstable thoracolumbar fractures. Mobile cone-beam CT (CBCT) systems with robotic image acquisition enable automated navigation workflows, but clinical data in spine trauma remain limited.

**Research question:**

To compare pedicle screw accuracy and perioperative outcomes between automated CBCT-based navigation and conventional fluoroscopy in thoracolumbar fracture fixation.

**Material and methods:**

This single-centre retrospective cohort study included adults with unstable thoracolumbar spine injuries undergoing pedicle screw fixation during a 5.5-year period. In the CBCT group, intraoperative 3D navigation was performed using an automated CBCT system. Perioperative and postoperative variables were recorded, and screw placement was assessed using the Gertzbein–Robbins classification. Outcomes were compared between groups.

**Results:**

Forty-six patients were included: 25 treated with automated CBCT-based navigation and 21 with conventional fluoroscopy. Among 313 evaluable screws, 96.8% were graded Gertzbein–Robbins A or B, and no grade E breaches were observed. In CBCT screws, no grade D or E breach occurred, and no screw-related early reoperations were required. Total operative time was longer with CBCT (154 vs. 119 min; p = 0.018), and blood loss was numerically higher (259 vs. 179 mL; p = 0.272), although operative time and blood loss per pedicle screw did not differ significantly between groups. Length of hospital stay and early clinical outcomes were comparable.

**Conclusion:**

Automated CBCT-based intraoperative 3D navigation provides high pedicle screw accuracy and comparable perioperative outcomes. CBCT-based navigation enables real-time 3D visualization and intraoperative verification, supporting its role as a reliable imaging modality in spine trauma surgery.

## Highlights

• Automated cone-beam CT (CBCT) enabled 3D navigation and implant verification.
• Clinically acceptable screw placement was 96.3% with CBCT navigation.
• No severe breaches or screw-related early reoperations occurred with CBCT.
• Total operative time was longer with CBCT, but normalized time was comparable.
• Early clinical outcomes were comparable with conventional fluoroscopy.

## 1 Introduction

Pedicle screw fixation is the cornerstone of surgical stabilization for thoracolumbar spine fractures, providing immediate biomechanical stability and enabling early mobilization. Accurate screw placement is essential to maintain construct integrity and to prevent neurological or vascular injury. Malpositioned screws may compromise fixation strength, cause pain, or necessitate revision surgery (Kim et al., 2015; Gertzbein and Robbins, 1990; Gautschi et al., 2011).

Traditionally, pedicle screws have been inserted under two-dimensional C-arm fluoroscopy. While this technique is widely available and familiar to most spine surgeons, it provides limited axial visualization and relies heavily on anatomical experience, image interpretation, and tactile feedback. The approach also exposes both the patient and operating room staff to ionizing radiation and offers no true three-dimensional control during instrumentation (Gelalis et al., 2012; Holly and Foley, 2003).

Intraoperative navigation was introduced to improve implant placement accuracy and address some of these limitations. Earlier systems used preoperative computed tomography (CT) with surface matching registration, where anatomical landmarks were manually matched to the imaging dataset. Although effective, this method was vulnerable to registration errors and workflow delays if patient positioning or spinal alignment changed intraoperatively (Holly and Foley, 2003; Holly et al., 2006).

The advent of intraoperative three-dimensional (3D) imaging and navigation has substantially advanced spinal instrumentation (Holly and Foley, 2003; Otomo et al., 2022). Intraoperative CT systems enable acquisition of high-resolution 3D datasets with automatic registration and real-time instrument tracking. This eliminates the need for manual surface matching, reduces registration error, and allows intraoperative verification of screw placement before wound closure (Otomo et al., 2022; Frisk et al., 2024; Kendlbacher et al., 2022).

More recently, a new generation of mobile cone-beam CT (CBCT) systems with automated image acquisition has been introduced, representing a further technological advancement in intraoperative spinal imaging (Frisk et al., 2024; Kendlbacher et al., 2022; Mandelka et al., 2022). Similar to established intraoperative CT platforms, CBCT-based systems provide high-resolution 3D imaging with automatic registration and allow operating room personnel to leave the operating room during image acquisition, thereby reducing occupational radiation exposure (Kendlbacher et al., 2022; Nottmeier et al., 2013). In contrast to larger or less flexible intraoperative imaging setups, mobile CBCT platforms may offer practical workflow advantages through robotic positioning, a smaller footprint, and easier integration into the operating room environment (Kendlbacher et al., 2022; Mandelka et al., 2022). This may allow efficient image acquisition with less disruption of the surgical field and facilitates repeated intraoperative imaging when needed. Immediate image reconstruction with inherent registration supports navigated instrumentation and real-time verification of implant positioning, contributing to a more standardized and reproducible surgical workflow (Frisk et al., 2024; Kendlbacher et al., 2022; Mandelka et al., 2022).

These advantages may be particularly relevant in trauma settings, where anatomy may be distorted and intraoperative decision-making is time sensitive. In thoracolumbar fractures, prone positioning, reduction maneuvers, ligamentotaxis, and instrumentation may alter the relationship between preoperative imaging and the surgical anatomy. The main novelty of automated CBCT-based navigation is therefore not navigation alone, but the integration of mobile intraoperative 3D imaging, automatic registration, navigated instrumentation, and implant verification in a single workflow.

Despite these technological advances, clinical data specifically evaluating automated CBCT-based workflows in thoracolumbar fracture surgery remain limited (Mandelka et al., 2022). Moreover, direct comparisons of intraoperative 3D imaging or navigation with conventional fluoroscopy in routine thoracolumbar trauma practice remain relatively scarce (Lu et al., 2020; Mandelka et al., 2023; Brunken et al., 2024).

The present study therefore aimed to compare pedicle screw placement accuracy and perioperative outcomes between automated CBCT-based intraoperative 3D navigation and conventional fluoroscopy in thoracolumbar fracture fixation at a tertiary spine trauma center.

## 2 Material and methods

### 2.1 Study design

This retrospective single-center cohort study included patients with unstable injuries of the thoracolumbar spine (T1–L5) treated with posterior pedicle screw fixation using either automated CBCT-based intraoperative navigation or conventional fluoroscopy. All consecutive cases operated between January 2020 and August 2025 at Stavanger University Hospital, a tertiary referral center for spine trauma, were reviewed.

The treatment group was defined according to the intraoperative imaging modality used for pedicle screw placement. Automated CBCT-based navigation was introduced in January 2023 and was compared with conventional fluoroscopy, the imaging modality applied during the first three years of the study period. The primary outcome was pedicle screw placement accuracy according to the Gertzbein–Robbins classification. Secondary outcomes included operative time, estimated blood loss, length of hospital stay, reoperation for screw repositioning, and postoperative infection.

Clinical data were obtained from electronic medical records, operative reports, anesthesia records, and radiological imaging systems. The study was approved by the institutional data protection officer as a quality assurance project (eProtokoll ID 4993). Consent to participate was waived according to the Norwegian Healthcare Personnel Act.

### 2.2 Study population

Adult patients aged 18 years or older with unstable injuries of the thoracolumbar spine (T1–L5) requiring posterior pedicle screw fixation were eligible for inclusion. Injuries were confirmed by preoperative computed tomography (CT) and, when indicated, magnetic resonance imaging (MRI) before surgery. Both minimally invasive and open approaches were included. Patients requiring additional posterior decompression were also included. Although eligible fracture levels were limited to T1–L5, fixation constructs could extend caudally to S1.

Patients were excluded from the screw-level accuracy analysis if postoperative or intraoperative cross-sectional imaging suitable for Gertzbein–Robbins grading was not available. These patients remained included in the patient-level demographic, perioperative, and clinical outcome analyses.

### 2.3 Surgical techniques

Intraoperative navigation based on automated 3D imaging with a robotic CBCT system (Loop-X, Brainlab AG, Munich, Germany) was introduced in January 2023. The CBCT system acquires a non-isocentric scan volume of 25 cm length in one spin with dynamic collimation for dose reduction and is integrated with the navigation platform (Curve, Brainlab AG, Munich, Germany).

All surgeons involved were board-certified neurosurgeons or orthopaedic spine surgeons specialized in spinal surgery and trained in both CBCT-guided and fluoroscopy-guided pedicle screw placement.

Patients were positioned prone on a radiolucent table with the abdomen free to minimize venous pressure. All operations were performed under general anesthesia with standard antibiotic prophylaxis, sterile preparation, and draping. Operative time was recorded from first skin incision to wound closure; in the CBCT group, this included the initial incision and placement of the reference frame, CBCT acquisition and registration, navigation, and the routine control scan performed after all screws had been inserted. Estimated blood loss was calculated from the total fluid volume in the suction canister after subtracting the irrigation fluid used during the procedure.

In the fluoroscopy group, pedicle screws were inserted using a conventional percutaneous technique guided by biplanar C-arm fluoroscopy. Entry points were localized using anteroposterior and lateral fluoroscopic views. Jamshidi needles were advanced through the pedicle under fluoroscopic control, and Kirschner wires were placed for cannulated screw insertion. Rods were inserted percutaneously through small fascial incisions and secured to the screw heads.

In the CBCT group, after sterile draping, the patient reference was fixed to the spinous process or ilium, and the Loop-X CBCT system was positioned using remote control before an automated intraoperative cone-beam CT scan was obtained. During image acquisition, the patient was held in apnea to minimize respiratory motion and ensure optimal image quality and navigation accuracy. All operating room staff left the operating room during image acquisition to avoid radiation exposure. In unstable fracture situations, the reference array could be repositioned and separate CBCT acquisitions and navigation registrations obtained above and below the fracture.

The dataset was automatically registered with the navigation platform, allowing real-time instrument and implant tracking. Pedicle trajectories were planned based on the acquired 3D dataset. Small skin incisions were made directly over the planned entry points, followed by blunt dissection down to the pedicle surface. A navigated drill guide was then used to create the pedicle channel, and K-wires were inserted through the guide to facilitate placement of cannulated pedicle screws. After all pedicle screws had been inserted, a routine intraoperative CBCT control scan was obtained to verify implant position before rod insertion and wound closure.

In patients treated with a minimally invasive approach who also required decompression, a separate midline incision was made after screw placement. Limited posterior decompression was then performed as indicated, depending on the extent and location of canal compromise.

Open fixation was performed in cases requiring significant fracture reduction. A midline incision was made, and the paraspinal muscles were dissected subperiosteally to expose the laminae and pedicles bilaterally. Screw placement then followed the same general steps as described above, using either fluoroscopic or navigated guidance depending on group allocation. Rods were contoured and secured after fracture reduction was confirmed.

### 2.4 Radiological assessment

Postoperative CT scans were used to evaluate pedicle screw positioning whenever available. In cases where postoperative CT was not performed, assessment was based on intraoperative CBCT scans when available. Screws without cross-sectional CT or CBCT imaging were excluded from the screw-accuracy analysis, as plain radiographs were not considered sufficient for Gertzbein–Robbins grading.

Fractures were classified according to the AO Spine Thoracolumbar Classification System (Vaccaro et al., 2013). For analysis, fractures were grouped by their predominant structural injury type. In cases with mixed morphology, the higher-grade component was used for analysis.

Each screw was classified according to the Gertzbein–Robbins grading system, a validated and widely used method for assessing pedicle screw placement accuracy in spinal instrumentation (Gertzbein and Robbins, 1990). Screw positions were graded as grade A, no cortical breach; grade B, breach less than 2 mm; grade C, breach of 2 to less than 4 mm; grade D, breach of 4 to less than 6 mm; and grade E, breach of 6 mm or more or screw completely outside the pedicle (Gertzbein and Robbins, 1990). Grades A and B were considered clinically acceptable.

All available cross-sectional images were assessed by a consultant neurosurgeon experienced in spinal instrumentation using the prespecified Gertzbein–Robbins criteria. The same evaluator and grading protocol were applied to both groups. CT-based assessment is the established reference method and has demonstrated high observer reliability when performed by trained readers (Yoo et al., 1997; Kosmopoulos et al., 2007).

### 2.5 Statistics

Variables recorded included age, sex, body mass index (BMI), neurological status according to the ASIA impairment scale, AO Spine fracture classification, surgical approach, additional decompression, number of pedicle screws, number of instrumented vertebrae, vertebral level of screw placement, operative time, estimated blood loss, operative time and blood loss per pedicle screw, length of hospital stay, postoperative infection, and reoperation for screw repositioning.

Continuous variables are presented as mean values with standard deviation (SD) and were compared between groups using independent-samples t-tests. Categorical variables are presented as counts and percentages and were analyzed using Pearson's chi-square test or Fisher's exact test for sparse 2 × 2 comparisons. Operative time and blood loss were additionally evaluated relative to the number of pedicle screws. The overall distribution of screws across thoracic, lumbar, and sacral regions was compared using the Fisher–Freeman–Halton exact test because of sparse sacral counts.

Missing data were handled by available-case analysis for each variable. Denominators are reported in the tables when they differ from the full cohort. No imputation was performed. Because of the retrospective and exploratory design, no formal sample size calculation was performed. Statistical significance was defined as p < 0.05, and all tests were two-sided.

## 3 Results

Forty-six patients were included: 25 were operated using CBCT-based intraoperative 3D navigation and 21 using conventional fluoroscopy. The mean age was 46.28 years, and 28 patients were male. Among 34 patients with available BMI data, mean BMI was 26.47 kg/m^2^ in the fluoroscopy group and 28.58 kg/m^2^ in the CBCT group (p = 0.262). ASIA grade was available for 44 patients, of whom 13 had neurological deficit classified as ASIA A–D. According to the AO Spine classification, B-type injuries were the most frequent, followed by A4, A3, and C injuries. The distribution of fracture types and injury severity was similar between groups. Patient demographics and injury characteristics are presented in Table 1.

Most operations were performed with a minimally invasive approach. Four patients underwent open fixation: three in the fluoroscopy group and one in the CBCT group. Twelve patients had additional decompression, including six patients in each group.

In total, 345 pedicle screws were inserted: 150 in the fluoroscopy group and 195 in the CBCT group. The mean number of instrumented vertebrae per patient did not differ significantly between groups (3.96 with CBCT vs. 3.57 with fluoroscopy; p = 0.197). Mean operative time was longer in the CBCT group than in the fluoroscopy group (154 vs. 119 min; p = 0.018). When expressed per pedicle screw, however, operative time did not differ significantly between groups (21.40 vs. 18.53 min; p = 0.336).

Screws were placed from T2 to S1. Of the 345 inserted screws, 185 (53.6%) were thoracic, 154 (44.6%) lumbar, and 6 (1.7%) sacral. The regional distribution did not differ significantly between groups (Fisher–Freeman–Halton exact test, p = 0.352; Table 2, Table 3) (see Table 4).

**Table 1:** Patient demographics and injury characteristics.

Variable	All patients (n = 46)	Fluoroscopy (n = 21)	CBCT (n = 25)	P-value
Age, years, mean (SD)	46.28 (19.18)	47.81 (19.67)	45.00 (19.07)	0.626
Male sex, n (%)	28 (60.9)	14 (66.7)	14 (56.0)	0.460
BMI, mean (SD)	27.65 (5.38)	26.47 (5.73)	28.58 (5.04)	0.262
Spinal cord injury, ASIA A–D, n (%)	13/44 (29.5)	7/20 (35.0)	6/24 (25.0)	0.469
ASIA grade, n (%)				
A	2/44 (4.5)	1/20 (5.0)	1/24 (4.2)	0.535
B	1/44 (2.3)	1/20 (5.0)	0/24 (0.0)	
C	4/44 (9.1)	1/20 (5.0)	3/24 (12.5)	
D	6/44 (13.6)	4/20 (20.0)	2/24 (8.3)	
E	31/44 (70.5)	13/20 (65.0)	18/24 (75.0)	
AO Spine classification, n (%)				
A3	5 (10.9)	4 (19.0)	1 (4.0)	
A4	9 (19.6)	2 (9.5)	7 (28.0)	
B1	10 (21.7)	6 (28.6)	4 (16.0)	
B2	16 (34.8)	7 (33.3)	9 (36.0)	
B3	3 (6.5)	1 (4.8)	2 (8.0)	
C	3 (6.5)	1 (4.8)	2 (8.0)	

**Table note:** Values are presented as mean (SD) or n (%) unless otherwise specified. Percentages in the fluoroscopy and CBCT columns are calculated using the number of patients with available data within each treatment group as denominator. BMI was available for 34 patients: 15 in the fluoroscopy group and 19 in the CBCT group. ASIA grade was available for 44 patients: 20 in the fluoroscopy group and 24 in the CBCT group. Continuous variables were compared using independent-samples t-tests. Categorical variables were compared using Pearson's chi-square test. Abbreviations: ASIA, American Spinal Injury Association; BMI, body mass index; CBCT, cone-beam computed tomography; SD, standard deviation.

**Table 2:** Distribution of inserted pedicle screws, perioperative parameters, and early postoperative outcomes.

Variable	All patients/screws	Fluoroscopy	CBCT	p-value
Pedicle screws, n	345	150	195	
Pedicle screws per patient, mean (SD)	7.50 (1.92)	7.14 (1.74)	7.80 (2.04)	0.251
Number of thoracic screws, n (%)	185 (53.6)	76 (50.7)	109 (55.9)	0.352
Number of lumbar screws, n (%)	154 (44.6)	70 (46.7)	84 (43.1)	
Number of sacral screws (S1), n (%)	6 (1.7)	4 (2.7)	2 (1.0)	
Open surgical approach, n (%)	4 (8.7)	3 (14.3)	1 (4.0)	0.318*
Additional decompression, n (%)	12 (26.1)	6 (28.6)	6 (24.0)	0.749*
Instrumented vertebrae per patient, mean (SD)	3.78 (1.01)	3.57 (0.87)	3.96 (1.10)	0.197
Operative time, min, mean (SD)	138.95 (49.04)	119.21 (49.69)	153.96 (43.75)	0.018
Operative time per pedicle screw, min, mean (SD)	20.16 (9.71)	18.53 (10.17)	21.40 (9.36)	0.336
Blood loss, mL, mean (SD)	223.56 (239.70)	179.25 (243.70)	259.00 (235.32)	0.272
Blood loss per pedicle screw, mL, mean (SD)	29.15 (30.39)	26.70 (35.89)	31.11 (25.79)	0.634
Length of hospital stay, days, mean (SD)	8.50 (9.85)	8.71 (11.43)	8.32 (8.53)	0.894
Length of hospital stay without SCI, days, mean (SD)	4.87 (2.39)	4.62 (2.57)	5.06 (2.31)	0.621
Reoperation with screw repositioning, n (%)	2 (4.3)	2 (9.5)	0 (0.0)	0.203*
Postoperative infection, n (%)	4 (8.7)	1 (4.8)	3 (12.0)	0.614*

**Table note:** Values are presented as mean (SD) or n (%) unless otherwise specified. Patient-level categorical percentages are calculated using the number of patients within each treatment group as denominator. Screw-region percentages are calculated using all inserted pedicle screws within each treatment group as denominator (150 fluoroscopy; 195 CBCT). The single p-value spanning the thoracic, lumbar, and sacral rows compares their overall distribution using the Fisher–Freeman–Halton exact test. Operative time was available for 44 patients: 19 in the fluoroscopy group and 25 in the CBCT group. Blood loss was available for 45 patients: 20 in the fluoroscopy group and 25 in the CBCT group. Length of hospital stay without SCI was calculated for 31 patients without spinal cord injury: 13 in the fluoroscopy group and 18 in the CBCT group. Continuous variables were compared using independent-samples t-tests. Categorical variables were compared using Pearson's chi-square test or Fisher's exact test when appropriate. *Fisher's exact test. Abbreviations: CBCT, cone-beam computed tomography; SCI, spinal cord injury; SD, standard deviation.

**Table 3:** Distribution of all inserted pedicle screws by vertebral level.

Vertebral level	All screws (n = 345)	Fluoroscopy (n = 150)	CBCT (n = 195)
Thoracic	185 (53.6)	76 (50.7)	109 (55.9)
T1	0 (0.0)	0 (0.0)	0 (0.0)
T2	6 (1.7)	2 (1.3)	4 (2.1)
T3	4 (1.2)	0 (0.0)	4 (2.1)
T4	6 (1.7)	2 (1.3)	4 (2.1)
T5	8 (2.3)	4 (2.7)	4 (2.1)
T6	8 (2.3)	2 (1.3)	6 (3.1)
T7	11 (3.2)	2 (1.3)	9 (4.6)
T8	14 (4.1)	6 (4.0)	8 (4.1)
T9	18 (5.2)	8 (5.3)	10 (5.1)
T10	20 (5.8)	8 (5.3)	12 (6.2)
T11	42 (12.2)	18 (12.0)	24 (12.3)
T12	48 (13.9)	24 (16.0)	24 (12.3)
Lumbar	154 (44.6)	70 (46.7)	84 (43.1)
L1	42 (12.2)	18 (12.0)	24 (12.3)
L2	42 (12.2)	16 (10.7)	26 (13.3)
L3	42 (12.2)	20 (13.3)	22 (11.3)
L4	24 (7.0)	14 (9.3)	10 (5.1)
L5	4 (1.2)	2 (1.3)	2 (1.0)
Sacral	6 (1.7)	4 (2.7)	2 (1.0)
S1	6 (1.7)	4 (2.7)	2 (1.0)
All levels	345 (100.0)	150 (100.0)	195 (100.0)

Table note: Values are presented as n (column percentage). The distribution includes all 345 inserted pedicle screws. Fracture levels were limited to T1–L5, but fixation constructs could extend caudally to S1. Abbreviation: CBCT, cone-beam computed tomography.

**Table 4:** Radiological grading of pedicle screw positions according to the Gertzbein–Robbins classification.

Variable	All screws (n = 313)	Fluoroscopy (n = 150)	CBCT (n = 163)	p-value
**Gertzbein–Robbins classification**				
Grade A	257 (82.1)	122 (81.3)	135 (82.8)	0.530
Grade B	46 (14.7)	24 (16.0)	22 (13.5)	
Grade A + B	303 (96.8)	146 (97.3)	157 (96.3)	
Grade C	9 (2.9)	3 (2.0)	6 (3.7)	
Grade D	1 (0.3)	1 (0.7)	0 (0.0)	
Grade E	0 (0.0)	0 (0.0)	0 (0.0)	

**Table note:** Values are presented as n (%). Percentages are calculated using the number of evaluable screws within each treatment group as denominator. Cross-sectional imaging suitable for Gertzbein–Robbins grading was available for 313 of 345 inserted pedicle screws: 150 of 150 screws in the fluoroscopy group and 163 of 195 screws in the CBCT group. Thirty-two CBCT screws were excluded from the screw-level accuracy analysis because Gertzbein–Robbins grading was not available. Gertzbein–Robbins grade A indicates no cortical breach, grade B a breach <2 mm, grade C a breach of 2 to <4 mm, grade D a breach of 4 to <6 mm, and grade E a breach ≥6 mm or screw completely outside the pedicle. Grades A and B were considered clinically acceptable. The p-value for Gertzbein–Robbins classification compares the overall distribution of grades A–D between groups using Pearson's chi-square test; grade E was not included because both groups had zero screws in this category. Clinically acceptable versus non-acceptable screw position, defined as Gertzbein–Robbins A + B versus C–E, was compared using Fisher's exact test and was not significantly different between groups (p = 0.752). Abbreviation: CBCT, cone-beam computed tomography.

Mean total blood loss was numerically higher in the CBCT group than in the fluoroscopy group (259 vs. 179 mL), but this difference was not statistically significant (p = 0.272). Blood loss per pedicle screw also did not differ significantly between groups (31.11 vs. 26.70 mL; p = 0.634).

Mean length of hospital stay was 8.50 days and did not differ significantly between groups. Length of hospital stay among patients without spinal cord injury was 4.87 days overall, with no significant difference between groups. Postoperative infection occurred in four patients, one in the fluoroscopy group and three in the CBCT group. In the fluoroscopy group, two reoperations involved repositioning of malpositioned screws confirmed on postoperative CT; no early reoperation for screw repositioning was required in the CBCT group.

Postoperative or intraoperative cross-sectional imaging suitable for Gertzbein–Robbins assessment was available for 313 of the 345 inserted pedicle screws (Table 4). Thirty-two screws in the CBCT group were not available for screw-level accuracy analysis because cross-sectional imaging suitable for Gertzbein–Robbins grading was not available. Of the evaluable screws, 150 were placed under fluoroscopic guidance and 163 using CBCT-based navigation. According to the Gertzbein–Robbins classification, 82.1% of screws were graded A, 14.7% grade B, 2.9% grade C, and 0.3% grade D; no grade E breaches were observed. The overall distribution of Gertzbein–Robbins grades did not differ significantly between groups (p = 0.530). Clinically acceptable placement, defined as grade A or B, was achieved in 96.8% of screws overall, with no significant difference between the fluoroscopy and CBCT groups (97.3% vs. 96.3%; p = 0.752). Among evaluable CBCT screws, no grade D or E breach occurred.

## 4 Discussion

This study found that automated CBCT-based intraoperative 3D navigation and conventional fluoroscopy both achieved high pedicle screw accuracy and comparable early safety in thoracolumbar fracture fixation. Among evaluable CBCT screws, no severe breach occurred, and no patient in the CBCT group required early reoperation for screw repositioning. Total operative time was longer with CBCT, whereas operative time per pedicle screw, blood loss, length of hospital stay, postoperative infection, and reoperation for screw repositioning were not significantly different between groups. Because operative time was measured from first skin incision to wound closure, the observed difference reflects the entire procedure rather than isolated screw-placement time and may partly reflect reference-array placement, the initial CBCT acquisition, automatic registration and navigation setup, and the routine control scan performed after all screws had been inserted. These findings suggest that automated CBCT-based imaging can be integrated into thoracolumbar trauma surgery without a measurable early clinical disadvantage, while acknowledging that the workflow may add time to the overall procedure.

Previous evidence is heterogeneous. Systematic reviews and meta-analyses have generally reported higher pedicle screw accuracy with navigated or robot-assisted techniques than with conventional fluoroscopic or freehand placement (Gelalis et al., 2012; Matur et al., 2023; Papalia et al., 2024), although a recent network meta-analysis found no statistically significant accuracy difference between navigated or robotic and conventional techniques (Riewruja et al., 2024). A large prospective randomized trial reported a significantly higher rate of clinically acceptable screw placement with robot-assisted navigation than with fluoroscopy (98.7% vs. 93.5%) (Han et al., 2019). At the level of individual comparative studies, however, superiority has not been demonstrated consistently. In a trauma-specific comparison, 3D navigation yielded numerically higher accuracy than fluoroscopy, but the difference was not statistically significant (92.7% vs. 88.1%; p = 0.19) (Brunken et al., 2024). A second thoracolumbar trauma cohort likewise found no significant difference in clinically acceptable screw trajectory between robotic navigation and fluoroscopy-guided percutaneous fixation (Katsevman et al., 2021), and a comparative series of 569 thoracic and lumbar screws found no significant accuracy difference among O-arm navigation, robotic guidance, and fluoroscopic freehand placement (Laudato et al., 2018). Taken together, the broader literature favors navigation overall while also showing that highly accurate fluoroscopic placement remains achievable in individual series. Our findings are consistent with this latter group of comparative studies. In our cohort, clinically acceptable placement was achieved in 97.3% of fluoroscopy-guided screws, providing a stringent comparator against which to demonstrate an incremental benefit, particularly in a modest sample. All procedures were performed by specialist spine surgeons experienced in conventional fluoroscopy-guided fixation, which may have contributed to the high comparator accuracy. Differences in case mix, surgeon experience, navigation platform, definitions of accuracy, and statistical power may explain why some studies demonstrate a clear benefit of navigation, whereas others report similarly high accuracy with both techniques. The absence of a statistically significant difference in our study should not be interpreted as evidence of equivalence or as contradicting the broader evidence favoring navigation. Rather, our findings demonstrate that conventional fluoroscopy can also achieve a high level of accuracy in experienced hands.

The present study adds trauma-specific data by comparing automated CBCT-based navigation directly with fluoroscopy in a cohort with high baseline fluoroscopic accuracy. Beyond the comparison of overall screw accuracy, the main potential value of CBCT lies in the integration of intraoperative 3D imaging, automatic registration, navigated instrumentation, and immediate implant verification, allowing decisions to reflect the patient's current operative anatomy and providing an opportunity to correct suboptimal implant positions before wound closure (Frisk et al., 2024; Kendlbacher et al., 2022; Mandelka et al., 2022; Vadalà et al., 2024; Zimmermann et al., 2021). This may be particularly useful in thoracolumbar trauma, where fracture displacement, positioning, reduction maneuvers, and progressive instrumentation can alter anatomic relationships, particularly in technically demanding situations such as thoracic or multilevel instrumentation, narrow pedicles, comminuted or translational injuries, obesity, and minimally invasive fixation. Automated CBCT-based navigation should therefore be viewed not simply as a replacement for fluoroscopy, but as a safe and precise workflow that adds intraoperative 3D control when anatomy is distorted or fluoroscopic assessment is challenging. Radiation exposure must also be considered from both patient and occupational perspectives. CBCT-based workflows may reduce occupational exposure because staff can leave the operating room during image acquisition, whereas patient dose depends on scan protocol, field of view, number of acquisitions, and the amount of fluoroscopy otherwise required (Nottmeier et al., 2013; Costa et al., 2016; Mason et al., 2024). Larger prospective studies should evaluate not only screw accuracy, but also workflow efficiency, radiation exposure to patients and staff, cost, revision surgery, postoperative imaging requirements, and long-term outcomes such as implant failure, fusion, pain, and functional recovery.

## 5 Limitations

This study has several limitations. It was retrospective, single-center, and non-randomized, and the CBCT group was compared with a partly historical fluoroscopy cohort. The sample size was modest, and the study was not powered to detect small differences in clinical complications or rare adverse events. Differences in case complexity, construct length, screw distribution across vertebral levels, learning curve, and temporal changes in surgical practice may have influenced operative time and other perioperative variables. Thirty-two screws lacked cross-sectional imaging suitable for Gertzbein–Robbins grading and were excluded from the screw-level accuracy analysis. Radiological assessment was performed by a single experienced assessor, and interobserver reliability was not evaluated. In addition, postoperative CT and intraoperative CBCT are not identical imaging modalities, which may influence detection of minor cortical breaches. Radiation dose metrics were not available, preventing direct comparison of patient and staff radiation exposure. Finally, the study focused on radiological accuracy and early postoperative outcomes; long-term outcomes such as implant failure, screw loosening, fusion, pain, and functional recovery were not assessed. These limitations mean that the results should be interpreted as supportive and hypothesis-generating rather than definitive evidence of superiority.

## 6 Conclusions

Automated CBCT-based intraoperative 3D navigation achieved high pedicle screw accuracy in thoracolumbar fracture fixation, with no early reoperations for screw repositioning in the CBCT group. Apart from longer total operative time, perioperative and early postoperative outcomes were comparable with conventional fluoroscopy. These findings support automated CBCT-based navigation as a precise and clinically useful imaging workflow in modern spine trauma surgery, while larger prospective studies are needed to clarify its impact on workflow, radiation exposure, cost, and long-term outcomes.

## Author contributions

K.A. and C.W. conceived and designed the study. K.A. collected the data and performed the statistical analyses. K.A. drafted the manuscript. D.W., M.B., C.A., K.H., E.F., and A.K.R. contributed to data interpretation and critically revised the manuscript for important intellectual content. C.W. supervised the study. All authors reviewed and approved the final manuscript.

## Data availability

The datasets are available from the corresponding author on reasonable request and in accordance with current General Data Protection Regulation (GDPR) guidelines.

## Ethical approval

This study was approved by the institutional data protection officer at Stavanger University Hospital as a quality assurance project (eProtokoll ID: 4993) according to the Norwegian Healthcare Personnel Act. Consent to participate was waived.

## Declaration of generative AI and AI-assisted technologies in the manuscript preparation process

During preparation of this work, the authors used ChatGPT for language editing and readability improvements only. The authors reviewed and edited the manuscript as needed and take full responsibility for the content of the final version.

## Funding

No funding was received for this study.

## Declaration of competing interest

The authors declare that they have no known competing financial interests or personal relationships that could have appeared to influence the work reported in this paper.
